# The Value of Changing Position in the Detection of CSF Leakage in Spontaneous Intracranial Hypotension Using Tc-99m DTPA Scintigraphy: Two Case Reports

**DOI:** 10.5812/iranjradiol.7956

**Published:** 2012-09-17

**Authors:** Yu Yu Lu, Hsin Yi Wang, Ying Lin, Wan Yu Lin

**Affiliations:** 1 Department of Nuclear Medicine, Taichung Veterans General Hospital, Taichung, Taiwan

**Keywords:** Intracranial Hypotension, Technetium, Magnetic Resonance Imaging, Scintigraphy

## Abstract

Radionuclide Cisternography (RNC) is of potential value in pointing out the sites of cerebrospinal fluid (CSF) leakage in patients with spontaneous intracranial hypotension (SIH). In the current report, we present two patients who underwent RNC for suspected CSF leakage. Both patients underwent magnetic resonance imaging (MRI) and RNC for evaluation. We describe a simple method to increase the detection ability of RNC for CSF leakage in patients with SIH.

## 1. Introduction

Spontaneous intracranial hypotension (SIH) is a condition which is caused by spontaneous spinal cerebrospinal fluid (CSF) leakage without a history of dural trauma, which results in low CSF pressure. The most common neurologic symptom caused by low CSF pressure is headache, which is often orthostatic, i.e., occurs or worsens in the upright position and improves when the patient is recumbent (1-5). Evidence of CSF leaks may be obtained by radionuclide cisternography (RNC) which is helpful in up to 20 percent of the patients who have non-diagnostic magnetic resonance imaging (MRI) results (1). Traditionally, the acquisition protocols of RNC are conducted in the supine position. We herein present two cases of SIH who underwent RNC in the lying position for hours followed by the upright position.

## 2. Case Presentation

### 2.1. Case 1

**Figure 1:**
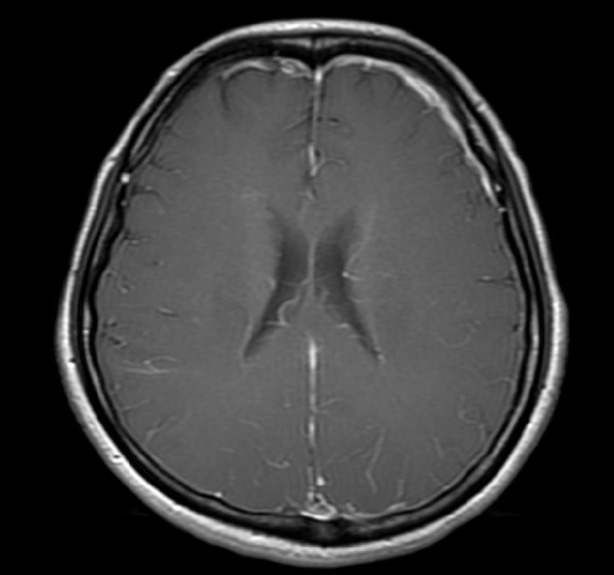
Axial T1-weighted view of cranial MRI after intravenous administration of gadolinium Pachymeningeal enhancement at the bilateral cerebral convexity with more irregular dural thickening at the left frontal convexity is demonstrated. This enhancement is due to small, thin-walled dilated blood vessels in the subdural zone

**Figure 2:**
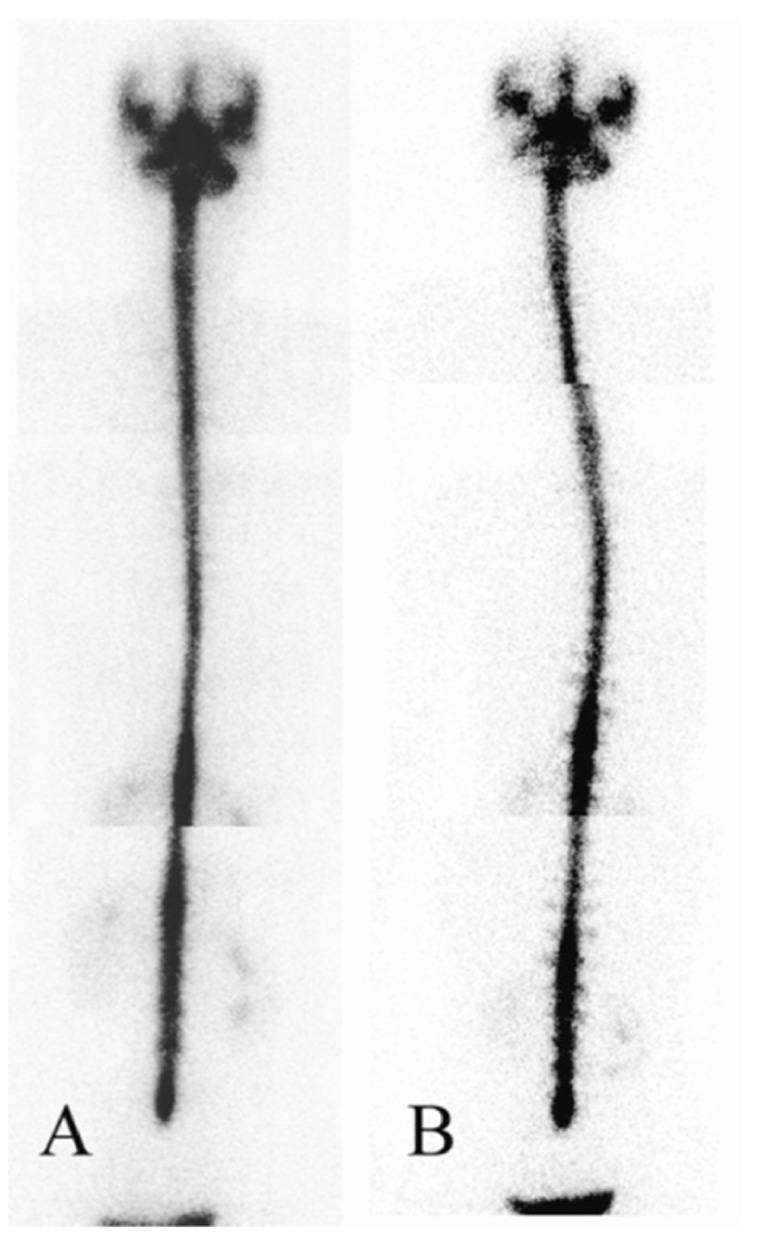
A 45-year-old woman underwent RNC for the possibility of CSF leakage Left column: Local images of the posterior views of the head, chest and abdomen were obtained at 6 hours in the supine position. Right column: Immediately after the 6-hour supine images, the posterior views obtained in the sitting position showed multiple areas of radioactivity on both sides of the paraspinal areas of the lumbar and lower thoracic levels

A 45-year-old woman had a sudden onset of severe headache over bilateral temporal and frontal regions for one week. Her headache worsened in the upright position and improved after lying down. Physical examination, routine blood tests and chest radiography on admission were normal. A CSF study after a lumbar puncture showed a CSF opening pressure of 3.5 cmH2O, 3 erythrocytes, 1 leukocyte, 1 lymphocyte/cumm, a protein level of 42 mg/dl (normal, 15 to 45 mg/dl) and a glucose level of 65 mg/dl. Results of all spinal fluid cultures were negative. Brain MRI showed no mass effect but pachymeningeal enhancement at the bilateral cerebral convexity with more marked enhancement at the left frontal convexity on contrast-enhanced T1 weighted study (Figure 1) was seen. SIH was impressed by the clinical physician. An RNC was performed after brain MRI by administering 170 MBq (4.6mCi) 99mTc-DTPA via lumbar puncture. Bed rest for six hours was ordered after the lumbar puncture to minimize post-puncture CSF leakage along the needle tract. Serial planar images of the whole body were obtained at 30 minutes and 1, 2, 3.5, and 6 hours after radioisotope injection. Local images of the posterior views of the head, chest and abdomen were obtained at 6 hours. All images were obtained in the supine position within 6 hours. Early appearance of urinary bladder on the 3.5-hour image was noted. However, direct evidence of extra-dural accumulation of radioactivity was not found. After the 6-hour images in the supine position were acquired, additional local images of the posterior views of the head, chest and abdomen in the upright position were also acquired. The images showed multiple areas of radioactivity on both sides of the paraspinal areas of the lumbar and lower thoracic levels (Figure 2) suggestive of CSF leakage. Epidural blood patch (EBP) using 12 ml of autologous blood injected into the epidural space of the L3-L4 level was performed because of failure of conservative treatment including bed rest, analgesic and hydration. The severity of her headache improved after this procedure.

### 2.2. Case 2

**Figure 3:**
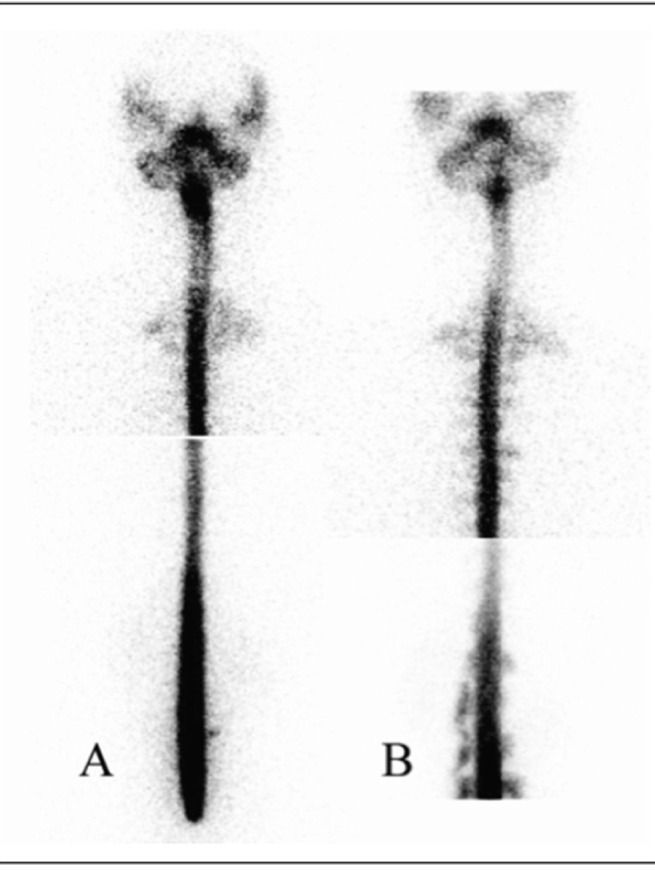
A 36-year-old woman underwent radionuclide cisternography to identify the site of CSF leakage. Left column: On 6-hour post-injection image, RNC showed areas of tracer accumulation along both sides of the paraspinal areas at the upper thoracic level and the right side of the lumbar level. Right column: Immediately after the 6-hour supine imaging, the posterior views obtained in the sitting position showed additional sites of CSF leakage at both sides of the paraspinal areas at the thoracic and lumbar levels, especially at the lumbar spine.

A 36-year-old woman was admitted with sudden onset of positional headache for 9 days. Her headache occurred when sitting up or standing up for 20-40 minutes and was relieved when lying down. The patient had no history of spinal trauma, including previous lumbar puncture, surgery or accident. Lumbar puncture and CSF study showed a CSF pressure of 0 mmH2O. Brain magnetic resonance imaging was negative. An RNC was performed using the same protocol mentioned above. Areas of tracer accumulation along both sides of the paraspinal areas at the upper thoracic level and lumbar level were demonstrated on 1-hour, 3-hour and 6-hour images. Immediately after the 6-hour supine imaging, the images taken in the sitting position showed clearer tracer accumulation along both sides of the paraspinal areas at the upper thoracic level and the right side of the lumbar level. Furthermore, additional sites of CSF leakage were identified at both sides of the paraspinal areas at the thoracic and lumbar levels, especially at the lumbar spine (Figure 3). EBP using autologous blood was performed. Her symptoms improved after management.

## 3. Discussion

RNC is of potential value in diagnosing CSF leakage for SIH.5 Extra-dural accumulation of radioactivity is a direct sign of CSF leakage (6, 7). In addition, early appearance of urinary bladder activity and slow ascent to the cerebral convexities, indicating absorption of tracer leakage through the dural defect into the bloodstream, can be considered as indirect signs of CSF leakage (4, 6-9). However, indirect signs in RNC are not reliable in diagnosing SIH.(10) Therefore, enhancing the appearance of the direct sign is a better way to increase the sensitivity of RNC without compromising the specificity. In the first case, the RNC only showed indirect findings of the early appearance of radioactivity in the urinary bladder and kidneys within 6 hours on serial images in the supine position. It is remarkable that simply by altering the acquisition position from supine to upright, multiple areas of increased radioactivity at the bilateral paraspinal areas of the lumbar and lower thoracic spines were demonstrated at the sixth hour after tracer administration. In the second case, RNC showed increased tracer accumulation along the bilateral paraspinal areas at the upper thoracic and lumbar levels before acquisition in the sitting position. Interestingly, additional sites of CSF leakage were identified at both sides of the paraspinal areas at the thoracic and lumbar levels, especially at the lumbar spine, in the sitting position images of the patient. One possible explanation for improvement of the detection rate of CSF leakage in this method is the effect of gravity. The possibility of radioisotope accumulation in the lumbar area in the sitting position may result in slow ascent of radioisotope to the upper spinal region and brain, which may decrease the detection rate at the upper spinal region. Therefore, we did not suggest the upright position at the beginning of this procedure. In our study, the patient remained in the supine position within the first 6 hours and then changed to the upright position for acquisition of a delayed image.

Potential technical errors during lumbar puncture may result from incomplete penetration of the subarachnoid space by the needle. The RNC images usually reveal intense and focal accumulation of radioisotope in the extra-spinal space at the lumbar level. In addition, non-visualization of the upper spinal region or slow ascent of radioisotope to the upper spinal region may also occur. In our two cases, smooth ascent of radioisotope from the lumbar area to the cerebral convexity and no obvious accumulation of radioisotope in the lumbar region were demonstrated which indicated a successful injection of the radioisotope via the lumbar puncture.

Complications from lumbar puncture include herniation, headache, cranial neuropathies, nerve root irritation, low back pain, infection and bleeding. The most life-threatening complication is herniation, which may result from a large pressure gradient between the cranial and lumbar compartments. Headache is the most common complication occurring in approximately thirty-six percent of patients within 48 hours after the procedure. Headaches may be caused by CSF leakage through the puncture site at a rate that exceeds the rate of CSF production.(11) Sufficient bed rest is recommended to minimize post-puncture CSF leakage along the needle tract. Therefore, the time point of six hours after tracer administration was chosen for optimal imaging of CSF leakage in the upright position.

Differential diagnosis in the conditions causing focal areas of radionuclide concentration or retention including radionuclide leakage following lumbar puncture or neurosurgical procedures, spinal meningeal diverticula (type I and type II = Tarlov’s cyst), CSF fistula, CSF leak, subdural hematoma, subdural hygroma, myelomeningoceles, congenital anomaly of meningoceles, congenital arachnoid cysts, Sturge-Weber syndrome and Dandy-Walker cysts.(12) Radiological cranial imaging and myelography may reveal an underlying anatomical defect causing the leak. In our study, cranial MRI has been performed to exclude the potential organic problem and cranial anatomical variants. A limitation of our case report is that myelography was not performed to exclude the potential anatomical variant in the spinal region. However, based on the history taking, sign and symptoms of the patients and improvement of the patient’s condition after epidural blood patching, SIH was still the first diagnosis.

To our best knowledge, there is no similar report about usage of the upright method to increase the detection of CSF leakage in SIH. In a case reported by Caroline et al.(13), extra-spinal radiotracer activity was noted posterior to the cervico-thoracic junction after lying supine for 6 hours, nevertheless this extra-spinal radiotracer activity disappeared shortly after the patient stood. The authors interpreted the disappearance of activity after standing as a direct sign of cerebrospinal fluid leakage. Apparently, the image findings were very different between Dr. Caroline’s case and ours after the patients stood up. In Dr. Caroline’s case, we thought that visualization of the extra-spinal radiotracer activity posterior to the cervico-thoracic junction was tracer leakage to the depending area. Then, disappearance of the extra-spinal radiotracer activity shortly after standing might be due to the minimal amount of CSF leakage that was soon absorbed. Therefore, we suggest that the lying position for hours followed by the upright position may be effective in detecting CSF leakage.

In conclusion, RNC is useful in diagnosing and managing SIH because it can identify the CSF leakage. We emphasize that hours in the lying position followed by the upright position may be effective in CSF leakage detection, which provides accurate diagnosis and proper treatment of SIH.
